# *De Novo* Assembly and Characterization of Fruit Transcriptome in Black Pepper (*Piper nigrum*)

**DOI:** 10.1371/journal.pone.0129822

**Published:** 2015-06-29

**Authors:** Lisong Hu, Chaoyun Hao, Rui Fan, Baoduo Wu, Lehe Tan, Huasong Wu

**Affiliations:** 1 Spice and Beverage Research Institute, Chinese Academy of Tropical Agricultural Science (CATAS), Wanning, Hainan 571533, China; 2 Key Laboratory of Genetic Resources Utilization of Spice and Beverage Crops, Ministry of Agriculture, Wanning, Hainan 571533, China; 3 Hainan Provincial Key Laboratory of Genetic Improvement and Quality Regulation for Tropical Spice and Beverage Crops, Wanning, Hainan 571533, China; National Key Laboratory of Crop Genetic Improvement, CHINA

## Abstract

Black pepper is one of the most popular and oldest spices in the world and valued for its pungent constituent alkaloids. Pinerine is the main bioactive compound in pepper alkaloids, which perform unique physiological functions. However, the mechanisms of piperine synthesis are poorly understood. This study is the first to describe the fruit transcriptome of black pepper by sequencing on Illumina HiSeq 2000 platform. A total of 56,281,710 raw reads were obtained and assembled. From these raw reads, 44,061 unigenes with an average length of 1,345 nt were generated. During functional annotation, 40,537 unigenes were annotated in Gene Ontology categories, Kyoto Encyclopedia of Genes and Genomes pathways, Swiss-Prot database, and Nucleotide Collection (NR/NT) database. In addition, 8,196 simple sequence repeats (SSRs) were detected. In a detailed analysis of the transcriptome, housekeeping genes for quantitative polymerase chain reaction internal control, polymorphic SSRs, and lysine/ornithine metabolism-related genes were identified. These results validated the availability of our database. Our study could provide useful data for further research on piperine synthesis in black pepper.

## Introduction

Black pepper, which is known as the “King of Spices”, is one of the most important and popular spices in the world. Black pepper is an important member of the Piperaceae family originating from the southwestern region of India, where its trade was initially restricted [1]. Black pepper, with its characteristic pungency and flavor, has been used as an ingredient in many food preparations for thousands of years. As one of the most extensively used spice in the world, black pepper is cultivated in tropical areas. The volume of black pepper trade reached 1.9 billion US dollars from a production of 4.6 × 10^5^ tons in 2012 (Food and Agriculture Organization data). Black pepper is used not only in human diet but also in other applications, such as medicine, preservative, perfumery, and insecticide, because this species exhibits antioxidant, anti-inflammatory, and anticancer properties [2–4].

The significant value of black pepper is due to the presence of piperidine alkaloids. Piperine (1-piperoylpiperidine) is a pungent, nitrogenous substance and major alkaloid in black pepper [5]. Piperine, which is utilized as a gustatory enhancer, is an effective agonist at the transient receptor potential cation channel subfamily V member 1 receptor, evoking a painful burning sensation sufficient to be a deterrent to most animals [6,7]. By contrast, dietary intake of piperine significantly stimulates the digestive enzymes of the pancreas and the intestines. As a result, digestive capacity is enhanced and gastrointestinal food transit time is shortened [8,9]. With unique taste and digestive function of piperine, black pepper has been considered as a popular food seasoning worldwide. In medical research, piperine has been validated in in vitro experiments to protect against oxidative damage by quenching reactive oxygen species and inhibiting lipid peroxidation [10,11]. Despite dietary and medical importance of piperine in black pepper, the underlying molecular mechanism remains unclear. Previous research showed that piperine is derived from the products of the primary metabolism of lysine/ornithine, which has been extensively studied in model plants [12,13]. However, further research on piperine biosynthesis mechanism in black pepper as a non-model plant is limited because of lack of an available molecular database.

As a non-model plant, black pepper does not have available genomic information. In the absence of a sequenced genome, de novo assembly of RNA-Seq is a cost-effective method to study the transcriptomes of most organisms [14,15]. Millions of short tags have been generated from RAN-Seq platform, such as Roche 454, Illumina Genome Analyzer, and Applied Biosystems SOLiD. After short tags are assembled using specific tools and algorithms, genome and transcriptome sequences are interpreted. Sequence platform, assembly tools, and special bioinformatics analysis can be applied to generate a complex technology system for RNA-Seq, which can identify transcript sequence polymorphisms, novel trans-splicing, and splice isoforms [16–18]. Compared with traditional approaches, RNA-Seq technologies provide highly specific and quantitative measurements.

In this study, a fruit transcriptome of black pepper was analyzed using Illumina HiSeq 2000 platform. Approximately 56 million clean reads were generated and 44,061 de novo assembled unigenes were obtained. The housekeeping gene, lysine/ornithine-related genes, and some polymorphic simple sequence repeat (SSR) primers were identified. These results, which constitute the first dataset of the sequence of the black pepper fruit, provide a useful gene library for black pepper molecular research.

## Materials and Methods

### Plant materials and nucleic acid isolation

Plant materials were obtained from 10 year-old black pepper cultivar (*Piper nigrum* L. cv. ‘Reyin No. 1’) with collecting permit in Spice and Beverage Research Institute of Chinese Academy of Tropical Agricultural Science, Wanning, Hainan, China. Eight fruits of black pepper were collected every month after pollination. Total RNA was isolated individually using TRIzol reagent (Life Technologies). The high quality RNA were obtained through twice Chloroform/Isoamyl alcohol (24:1) purification, and then mixed with approximately the same quantity. Total 5μg mRNA was purified using poly-T oligo-attached magnetic beads (Life Technologies) for RNA-Seq. Different tissues of black pepper were collected (root, stem, leaf, flower, and fruits). Total RNA was also isolated from each tissue for further expression analysis.

### Illumina cDNA library construction and sequencing

An Illumina HiSeq 2000 library was constructed for Solexa sequencing. The enriched poly(A) mRNA was fragmented into small pieces of 200 bp to 700 bp by using divalent cations at 75°C. First-strand cDNA was synthesized by reverse transcriptase with random hexamer primers. Second-strand cDNA was subsequently synthesized by DNA Polymerase I and RNase H (Invitrogen). The cDNA fragments were ligated to sequencing adapters and analyzed through agarose gel electrophoresis to select suitable fragments for enrichment by polymerase chain reaction (PCR) amplification. The resulting cDNA library was sequenced on Illumina HiSeq 2000 platform in Beijing Genomics Institute Genomic Center in Shenzhen, China (http://www.genomics.cn).

### Sequence assembly and functional annotation

Total raw reads from sequencing were preprocessed to remove dirty raw reads, including (1) adapters that were added for reverse transcription and sequencing, (2) sequences with unknown nucleotides larger than 5%, and (3) low-quality reads (the rate of reads with quality value of ≤10 is more than 20%). The filtered clean reads were then assembled using Trinity method (http://trinityrnaseq.sourceforge.net/) [14]. Overlapping information in the short reads was used to construct contigs with high coverage. Afterward, the reads were mapped back to the contigs to connect these contigs and to identify the sequences that cannot be extended on either end. Such sequences were defined as unigenes. Optimal results were selected on the basis of evaluation results of the assembly encompassing the total number of unigenes, the distribution of unigene length, the N50 statistic, and the average coverage.

We conducted a BLAST search against the non-redundant protein (NR) and nucleotide sequences (NT) databases in the National Center for Biotechnology Information (NCBI), Swiss-Prot, and Clusters of Orthologous Groups (COG) with an *E* value cutoff of 10^−5^ to assign putative functions to the unigenes. The Gene Ontology (GO) and Kyoto Encyclopedia of Genes and Genomes (KEGG) classifications were conducted by using Blast2GO according to Gotz et al. [19]. Gene names were assigned to each unigene based on the best hit (highest score).

### Internal control gene identification

The housekeeping genes were identified from transcriptome data. The housekeeping genes were searched through gene descriptions in functional annotation based on the candidate gene name. The first-strand cDNA template of different tissues (root, stem, leafy, flower, and fruits) was synthesized using a reverse transcriptase kit (Thermo) in accordance with the manufacturer’s instructions. Quantitative reverse transcription-polymerase chain reaction (qRT-PCR) was performed using ABI 7500 Real-time PCR system (Applied Biosystems, Foster City, CA, USA) in accordance with the manufacturer’s instructions. For each analysis, qRT-PCR assays were conducted in three sample replications. Error bars denoted the standard error of three replications.

For internal control gene identification, Microsoft Excel file of raw expression values for the tested genes in different samples was imported into geNORM to analyze gene expression stability [20]. The specificity of amplifications was verified by melting curve analysis (60°C to 95°C) after 40 cycles.

### SSR detection

SSR was detected with MIcroSAtellite software in which unigenes were used as reference data. SSRs with length of more than 150 bp on both ends of the unigene were retained, and these sequences were used to design primers in Primer Premier 6.0 (PREMIER Biosoft International, Palo Alto, CA, USA). The primers were filtered by removing the following: (1) primers without SSRs; (2) primers aligned to unigene sequences with more than three mismatches in the 5′ site and one mismatch in the 3′ site; (3) primers aligned to more than one unigene.

Twelve Piperaceae species were used in polymorphism analysis (S4 Table). DNA was isolated according to the cetyl trimethylammonium bromide protocol (CTAB). PCR was conducted to determine the polymorphism of primers. The following thermal cycling conditions were applied: 95°C for 5 min and 35 cycles of 95°C for 60 s, 55°C for 30 s, and 72°C for 90 s. The PCR product was analyzed by denaturing polyacrylamide gel electrophoresis.

## Results

### Sequencing and de novo transcriptome assembly

The fruits of black pepper (*P*. *nigrum* L. cv. Reyin No. 1) at different developmental stages were collected at 1 month to 10 months post anthesis. The RNA of these fruits was isolated. RNAs of equal quality were mixed for Illumina sequencing. A total of 56,281,710 raw reads were obtained. We filtered the sequence data for clean reads, resulting in 52,098,738 clean reads. All clean reads were de novo assembled into contigs by using the Trinity method [14]. The clean read assembly generated 179,075 contigs when all isoforms were included. These contigs represent a total of 44,061 unigenes that were considered for downstream analysis with an N50 length of 1,757 nt. The length of the unigenes ranged from 300 nt to 15,000 nt, with a mean length of 1,354 nt and 46.60% GC content (Table 1). A total of 23,085 (52.39%) unigenes longer than 1 kb and 9,769 (22.17%) unigenes longer than 2 kb were obtained. The length distributions of the unigenes are shown in Table 1; the results revealed that more than 30,000 unigenes (75.77%) were longer than 500 nt (Table 1). The database was deposited in the NCBI Sequence Read Archive (http://www.ncbi.nlm.nih.gov/Traces/sra/) under accession number SRS856941.

**Table 1:** Summary of transcriptome data for black pepper fruits.

	Number	Percentage
Raw reads	56,281,710	
Clean reads	52,098,738	
Contigs	179,075	
Total unigenes	44,061	
Total sizes (nt)	59,262,045	
Unigenes (300–500 nt)	10,678	24.23%
Unigenes (500–1000 nt)	10,298	23.38%
Unigenes (1000–2000 nt)	13,316	30.22%
Unigenes (2000–5000 nt)	9,409	21.35%
Unigenes (>5000 nt)	360	0.82%
Mean length (nt)	1,345	
GC content N50 length	1,757	46.60%
Unigenes in NR database	32,697	74.20%
Unigenes in NT database	25,366	57.57
Unigenes in SwissProt database	23,080	52.38%
Unigenes in GO database	28,827	65.43%
Unigenes in COG database	16,195	36.76%
Unigenes in KEGG database	24,836	56.37%

### Gene annotation and functional classification

**Fig 1:**
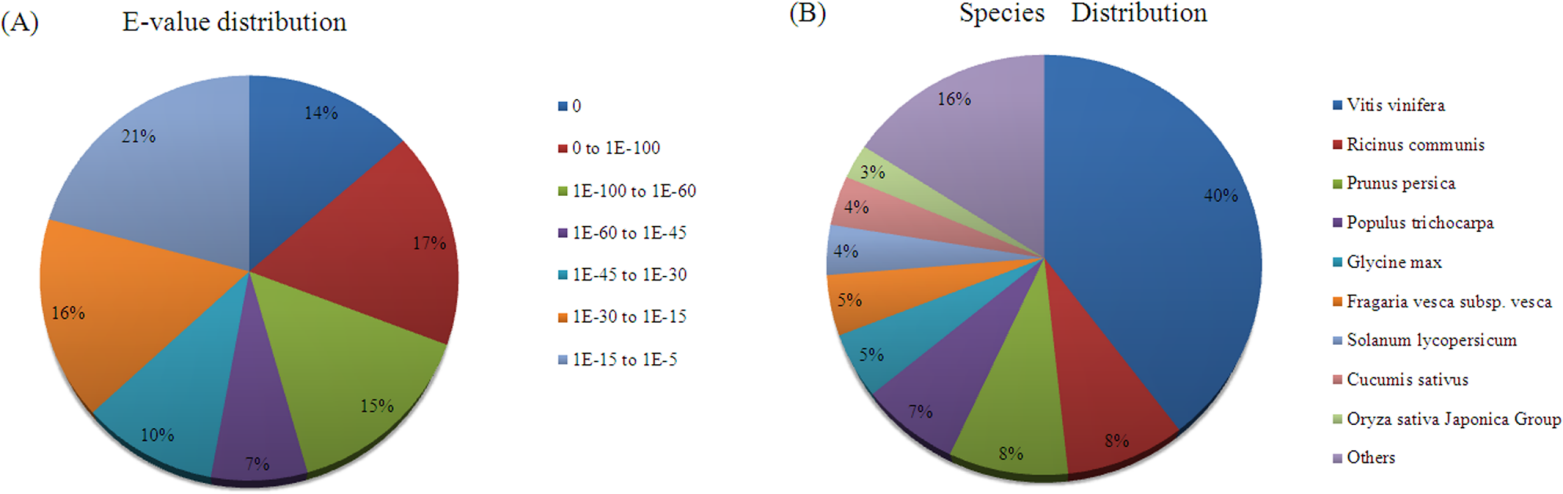
Summary results of NR sequence annotation. (A) *E* value distribution of BLASTX matches for each unigene. (B) Species-based distribution of BLASTX matches for each unigene.

The homology-based approach was adopted in functional annotation. All unigenes were searched against the NCBI NR and NT databases through BLASTX searches in which an *E* value cutoff of 10^−5^ was set. A total of 32,697 (74.20%) unigenes showed significant similarity to known proteins in the NR database. Likewise, a total of 25,366 (57.57%) exhibited significant similarity to known nucleotide sequences in the NT database. Sequences were also searched against the Swiss-Prot database to detect additional reference annotations, resulting in 23,080 (52.38%) annotated unigenes (Table 1). In the analysis of the matching sequences based on NR annotation, *E* value distribution showed that 55.80% of the annotated sequences displayed strong homology (*E* value less than 1*E* − 45; Fig 1A). In species distribution analysis, *Vitis vinifera* was ranked first with 12,948 (39.68%) top BLAST hits, followed by *Ricinus communis*, *Prunus persica*, *Populus trichocarpa*, and *Glycine max* with 2,813 (8.62%), 2,809 (8.60%), 2,332 (7.15%), and 1,632 (5%) top BLAST hits, respectively (Fig 1B).

**Fig 2:**
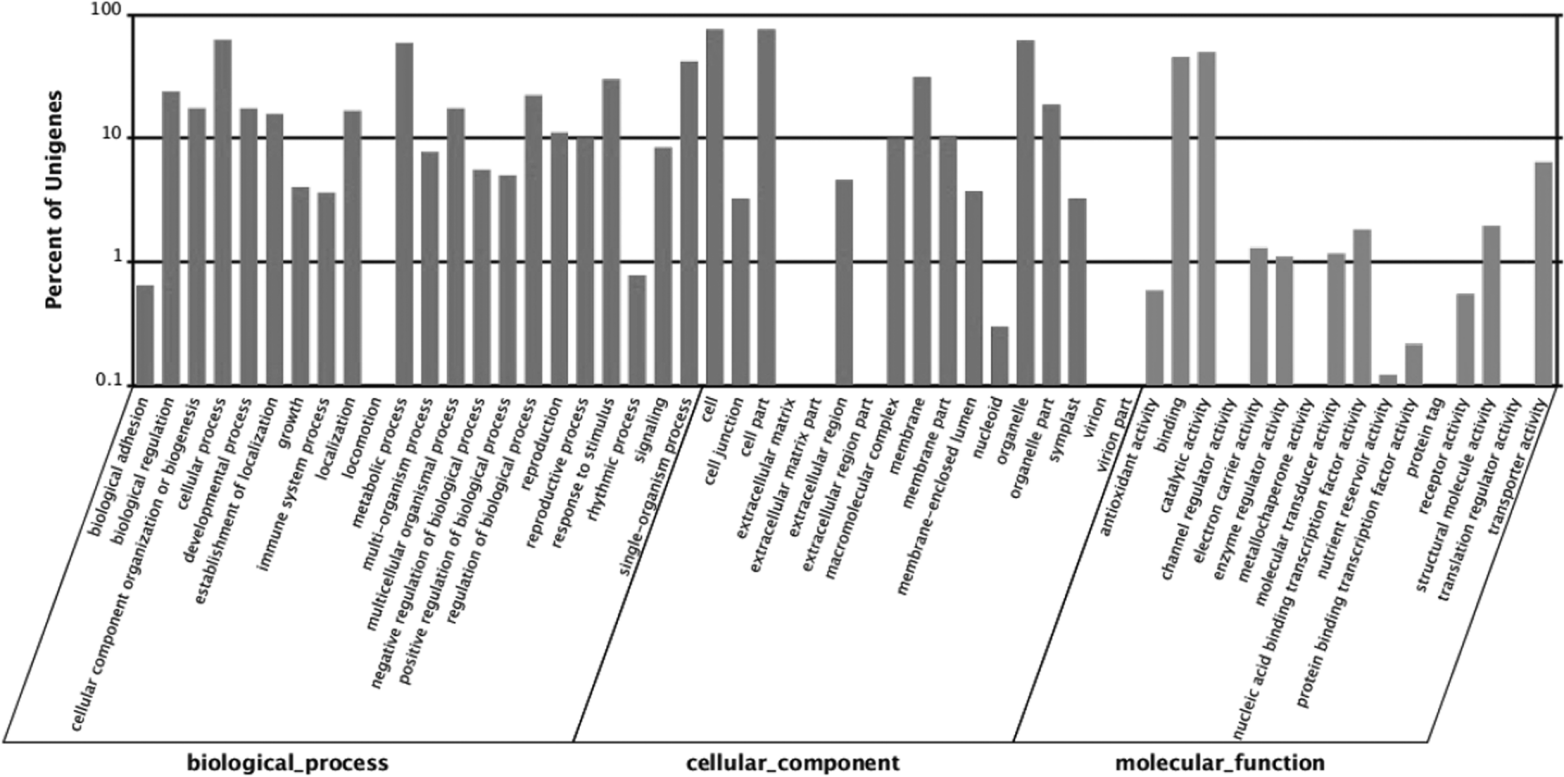
Distribution of GO classification (Level 2). Annotated unigenes were classified into 3 major categories (biological processes, cellular components, and molecular function) and 54 subgroups. The *x*-axis indicates the subgroups in GO annotation. The *y*-axis indicates the percentage of specific categories of genes in each main category.

**Fig 3:**
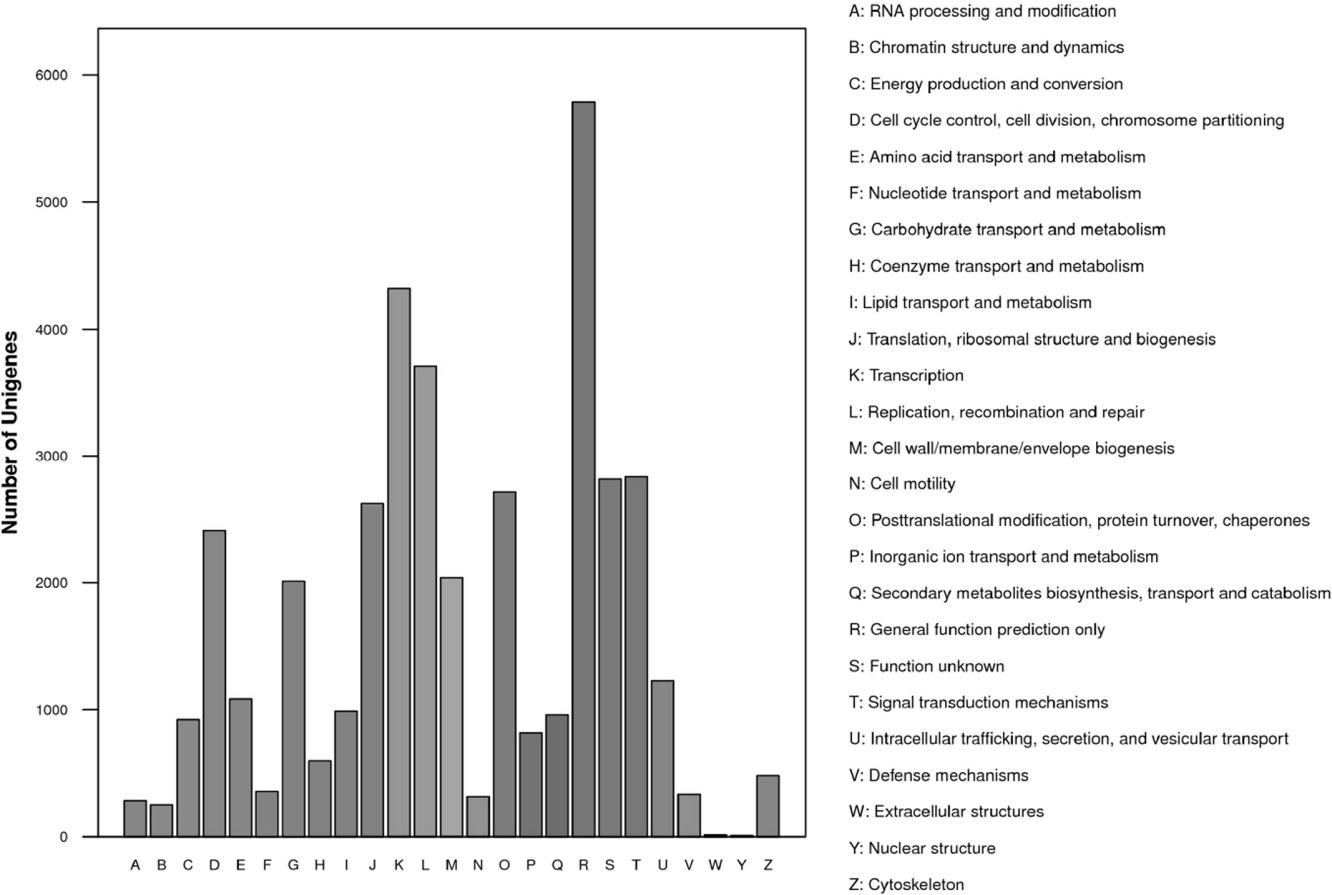
Distribution of COG classification. The unigenes with significant homologies in the COG database were classified into 25 COG categories. The *x*-axis indicates the subgroups in the COG classification. The *y*-axis indicates the number of genes in each main category.

As the international standardized gene functional classification system, GO and COG classifications were conducted for transcriptome data annotation. A total of 28,827 (65.43%) unigenes were assigned to 54 Level 2 GO terms, which were summarized under three main GO categories, including biological process, cellular component, and molecular function (Fig 2). Among all of the categories, cellular processes and metabolic processes in the biological processes, cell and cell part in the cellular component, and binding and catalytic activity in the molecular function represented the major subcategories. In the COG classification, 16,195 unigenes were classified into 24 COG categories. At the top, the clusters included general function prediction only, replication, and transcription (Fig 3).

All of these functional annotation assignments provide valuable information to investigate specific biochemical and developmental processes in fruit development of black pepper. The entire annotation information of transcriptome data is shown in S1 Table.

### KEGG pathway enrichment and piperine-related gene scanning

KEGG is a pathway-based categorization of orthologous genes that provide information to predict the functional profiles of genes. In this study, 24,836 genes were mapped into 128 signaling pathways to further validate the biological pathways that are active in the black pepper fruit. Among these genes, 6,558 (26.4%) unigenes were involved in the metabolic pathway, which was the main group in pathway enrichment, followed by biosynthesis of secondary metabolites (2,399; 9.7%), endocytosis (1,772; 7.1%), and glycerophospholipid (1,711; 6.9%) (S2 Table). These results provided a valuable resource that could be used to investigate specific processes and pathways in the development of black pepper fruit.

A previous study showed that piperidine alkaloids are derived from the products of lysine/ornithine metabolism [21]. The unigenes with full functional domain, annotated in the lysine/ornithine metabolism-related pathway, were identified manually. A total of 17 typical genes were identified as potentially related to lysine/ornithine metabolism, including lysine/ornithine decarboxylase, lysine dehydrogenase, and primary amine oxidase (Table 2). These genes might participate in piperidine, quinolizidine, indolizidine, and lycopodium alkaloid biosynthesis, which will provide valuable resource for further research.

**Table 2:** The list of lysine mechanism-related genes in transcriptome.

Gene ID	Length	KO_id	ko_definition
CL3230.Contig3_F0463-PN	1711	K01581	ornithine decarboxylase [EC:4.1.1.17]
CL1221.Contig2_F0463-PN	1806	K01586	diaminopimelate decarboxylase [EC:4.1.1.20]
CL16778.Contig2_F0463-PN	1610	K01581	ornithine decarboxylase [EC:4.1.1.17]
CL44.Contig2_F0463-PN	2878	K00276	primary-amine oxidase [EC:1.4.3.21]
CL9334.Contig1_F0463-PN	2082	K13367	non-specific polyamine oxidase [EC:1.5.3.17]
Unigene14041_F0463-PN	2504	K00276	primary-amine oxidase [EC:1.4.3.21]
CL13005.Contig1_F0463-PN	1978	K00818	acetylornithine aminotransferase [EC:2.6.1.11]
Unigene14591_F0463-PN	2315	K00276	primary-amine oxidase [EC:1.4.3.21]
Unigene7223_F0463-PN	2206	K00276	primary-amine oxidase [EC:1.4.3.21]
CL9890.Contig2_F0463-PN	1293	K01778	diaminopimelate epimerase [EC:5.1.1.7]
CL3253.Contig4_F0463-PN	1552	K13065	shikimate O-hydroxycinnamoyltransferase [EC:2.3.1.133]
CL3823.Contig1_F0463-PN	1696	K01438	acetylornithine deacetylase [EC:3.5.1.16]
CL3485.Contig1_F0463-PN	2233	K00928	aspartate kinase [EC:2.7.2.4]
CL13479.Contig1_F0463-PN	6480	K11446	histone lysine-demethylase JARID1 [EC:1.14.11.-]
CL5146.Contig2_F0463-PN	3370	K15601	lysine-specific demethylase 3 [EC:1.14.11.-]
CL14382.Contig2_F0463-PN	1349	K00215	dihydrodipicolinate reductase [EC:1.3.1.26]
CL1221.Contig2_F0463-PN	1806	K01586	diaminopimelate decarboxylase [EC:4.1.1.20]

### Internal control gene identification

**Fig 4:**
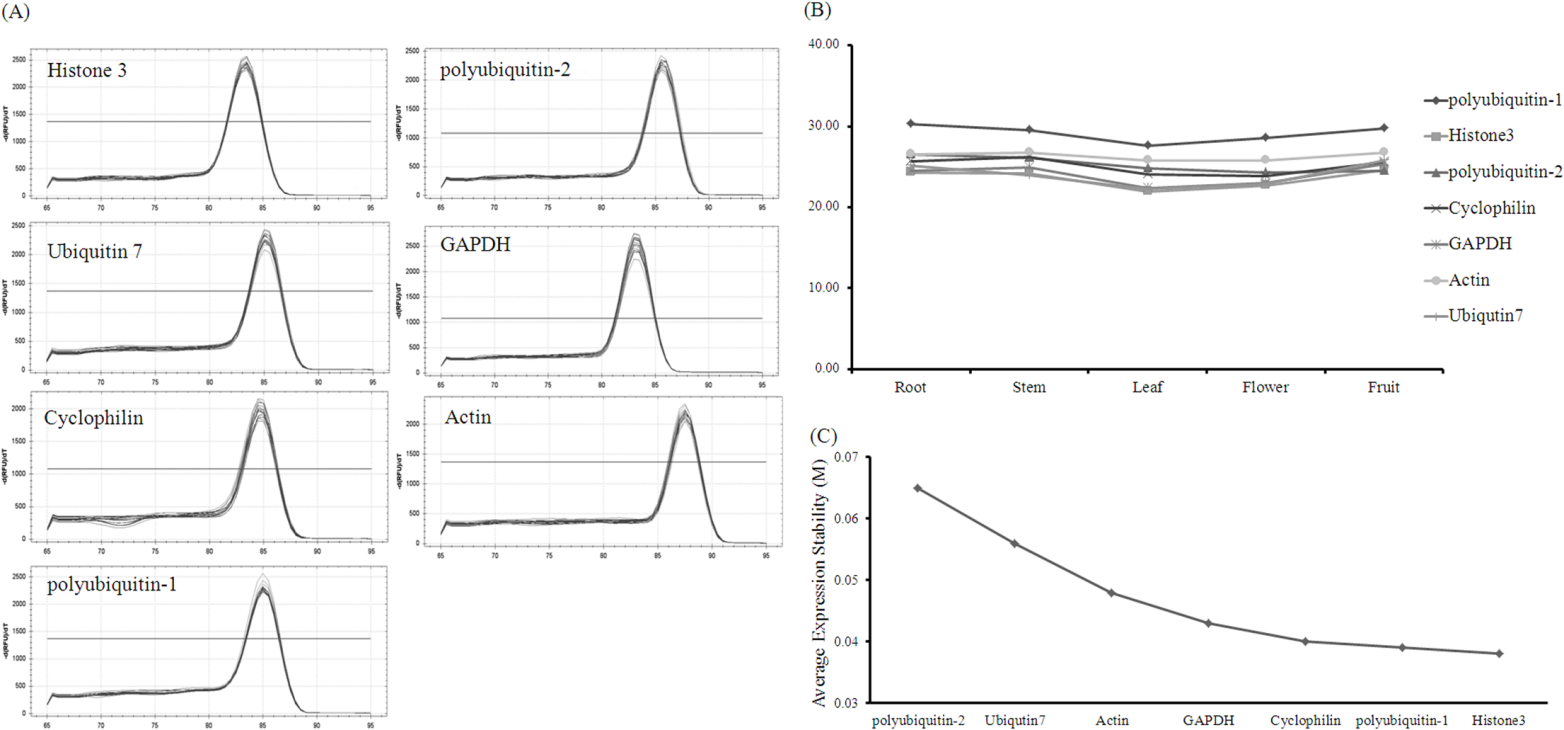
Identification of internal control genes. (A) Dissociation curves of seven genes in different samples. One specific peak represents the specific amplification of genes. (B) *C*_*t*_ values of genes in different samples. (C) Average expression stability values of genes, in which the lowest mean *M* value of histone H3 indicates the most stable expression.

Internal control genes were utilized for further analysis to assess transcriptome quality and provide fundamental basis for specifically expressed gene scanning. In this study, seven frequently used internal control genes were selected from the transcriptome annotation data by using key word search. These internal control genes were histone H3, ubiquitin-7, cyclophilin, polyubiquitin-1, polyubiquitin-2, glyceraldehyde-3-phosphate dehydrogenase gene (GAPDH), and actin. The primers were designed with amplification lengths of 100 nt to 200 nt and melting temperatures (*T*_*m*_) of 55°C to 60°C (Table 3). The expression stabilities of these seven genes were assessed by two-step real-time PCR in a set of five different tissue samples. The dissociation curves showed one specific peak, which indicated the specific amplification of PCR (Fig 4A). The cycle threshold (*C*_*t*_) values of these seven internal control genes in different tissues were also obtained through real-time PCR amplification, which varied not so obviously (Fig 4B). Then, geNORM was applied to identify the best internal control genes in various tissue samples. geNORM is a statistical algorithm that determines gene stability measure (*M*) of all of the genes under investigation based on the *C*_*t*_ value in different samples; a lower *M* value corresponds to a more stable expression [20]. After *C*_*t*_ value was evaluated, the results showed that the average expression *M* value of histone H3 was the lowest and most suitable internal control gene for black pepper (Fig 4C). Therefore, histone H3 can be selected as a reference gene for tissue-specific gene analysis.

**Table 3:** The primers of internal control genes for real-time PCR analysis.

Gene name	Gene NO.	Primers (5`-3`)
Histone 3	CL15850.Contig2_F0463-PN	GAAGTCAGCCCCGACGACAGGT
		CGAACGAGACGCTGGAAAGGTA
Ubiquitin 7	Unigene17089_F0463-PN	CCCCAGACCAGCAGCGTTTAATC
		CATCGACCTTGTAGAACTGCAGGAC
Actin	CL6615.Contig1_F0463-PN	GAAACTGGGTATCTGTGAGGCTGA
		GCAAGTGCTTCCTGATGAACAACA
GAPDH	CL9041.Contig3_F0463-PN	CTTTCTGTAGCCAACTCCTCTCTCC
		GACGGAGAAGAAGTCATCGGAAG
Cyclophilin	Unigene5924_F0463-PN	CCATTTGTGTCAGACCCAGCATT
		GGTGATGGTAGAGGAGGGGAGTC
Polyubiquitin-1	Unigene8228_F0463-PN	TTACCAGGACTCAGCAGCGAATG
		AAGCCAATGACTTTACATCCTCCAG
Polyubiquitin-2	CL13523.Contig3_F0463-PN	AGGAACGAGTTGAAGAGAAAGAAGG
		TCCACCCCGTAGAGCCAGAACAAG

### Detection of SSR markers

**Fig 5:**
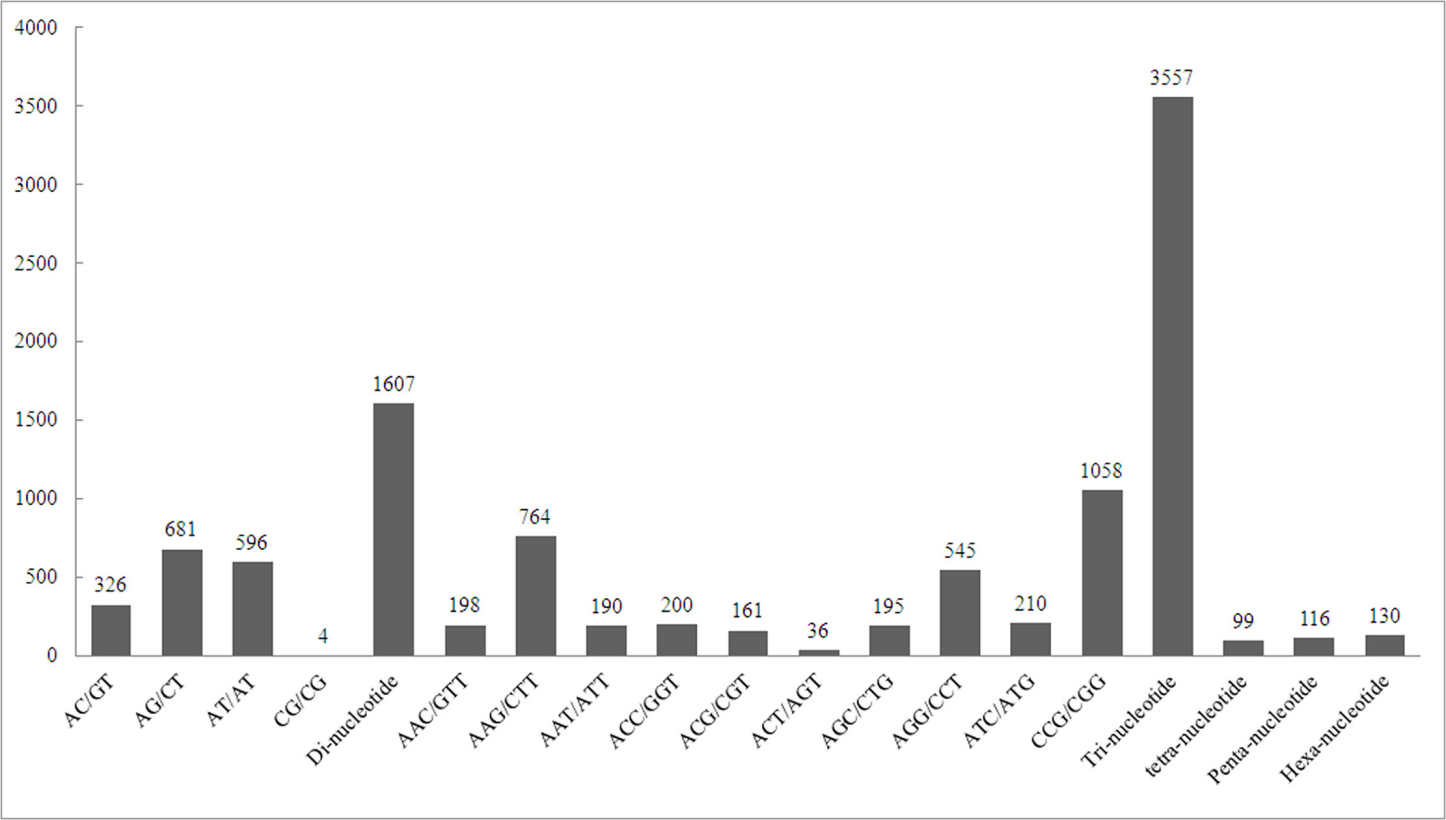
Summary of the SSR types in the transcriptome. A total of 5,509 SSRs were identified. The *x*-axis indicates the repeat type. The *y*-axis indicates the number of different repeats.

SSR served as the most important molecular marker, which has been extensively utilized for gene mapping, molecular breeding, genetic diversity, and discrimination. A total of 5,509 SSRs loci with dinucleotide, trinucleotide, tetranucleotide, pentanucleotide, and hexanucleotide repeats were detected in 5,252 unigenes by scanning the transcriptome data. Among these SSRs, the trinucleotide repeat motifs were the most abundant, accounting for 3,557 SSRs (64.57%), followed by 1,607 (29.17%) dinucleotide repeat motifs, 130 (2.36%) hexanucleotide repeats, 116 (2.10%) pentanucleotide repeat motifs, and 99 (1.80%) tetranucleotide repeat motifs (Table 4). The main motifs were the dinucleotide AG/CT repeat (681) and AT/TA repeat (596) and the trinucleotide CCG/CGG repeat (1,058) and AGG/CCT repeat (545). Detailed information of the SSR type is shown in Fig 5.

**Table 4:** Summary of SSR searching results.

Searching Item	NO.
Total number of unigenes examined	44,061
Total size of examined sequences (nt)	59,262,045
Total number of identified SSRs	5,509
Number of SSR containing unigenes	5,252
Number of sequences containing more than 1 SSR	217
Di-nucleotide	1,607
Tri-nucleotide	3,557
Tetra-nucleotide	99
Penta-nucleotide	116
Hexa-nucleotide	130

Based on the SSR-containing unigenes, the SSR primers were designed using Primer Premier 6.0 (PREMIER Biosoft International, Palo Alto, CA, USA) according to Wang et al. [22]. In total, 3,681 pairs of primers were obtained (S3 Table). A total of 24 pairs of primers were randomly selected for PCR amplification, and 12 Piperaceae species were selected for polymorphism analysis of the primers. The results showed that 11 pairs of primers exhibited ideal polymorphisms (S4 Table). These SSR primers could represent a valuable biomarker resource of *P*. *nigrum*. However, all putative SSR primers should be validated before use.

## Discussion

As one of the most popular and oldest spices, black pepper has been used in diet, perfumery, and medicine for thousands of years [23]. However, molecular biology research and available gene data for molecular research are limited [24–27]. Nevertheless, high-throughput RNA-Seq is an effective method to obtain large amounts of transcriptome data from different tissue types. In this study, the transcriptome of black pepper fruit was described for the first time. High-quality transcriptome data were obtained using Illumina HiSeq 2000 sequencing platform and assembled using the Trinity method with multiple optimal *k*-mer lengths and cutoff values. The assembly strategy was considered as a unified solution for transcriptome construction in any sample, particularly in the absence of a reference genome [14,28,29]. In sequence assembly, the N50 length was used to evaluate assemblies in which a high number corresponds to high quality. The high quality of our data was confirmed by high value of N50 and average length in sequence assembly (N50 = 1,575 bp, average length = 1,345 bp); this result was comparable to that obtained in published transcriptomic analyses of other plant species, such as *Reaumuria soongorica* (N50 = 1,109 bp, average length = 677 bp), *German cockroach* (N50 = 792 bp, average length = 798 bp), and *Haloxylon ammodendron* (N50 = 1,345 bp, average length = 728 bp) [30–32].

Functional annotation and classification were introduced to illustrate the transcriptome comprehensively. A large number of unigenes were annotated with molecular function by searching against the NCBI NR and NT and Swiss-Prot databases. Moreover, the annotated genes of black pepper showed higher homology to *V*. *vinifera* (Fig 1). The species distribution in annotation might reveal the evolutionary relationship of black pepper with other species. With specific metabolic alkaloids, black pepper has been considered as a common species among spices [10]. GO and COG classifications provided further insights into the role of metabolic alkaloids in the development of black pepper fruit. The main subcategories of gene distribution in cellular component and molecular function of GO annotation were similar to those of other species. In biological process, the high proportion of metabolic and cellular processes indicated the unique characteristic of black pepper development (Fig 2) [32,33].

Although RNA-Seq technology is an efficient method to describe the transcriptomes, further experiments were required to verify the utility of data. In this study, we focused on internal control gene identification in transcriptome data. An appropriate internal control should be determined to standardize the quantitative expression of different genes. Housekeeping genes, such as actin, tubulin, histone, and GAPDH, were most frequently applied for their stable expression in different tissues and conditions [34–36]. However, the transcript levels of these genes are not always stable. The suitable internal control genes for new species must be determined before use. Statistical algorithms, such as geNORM or BestKeeper, have been developed to determine the best suitable genes [20,37]. Housekeeping genes in our transcriptome data were searched by gene annotation. Seven sequences with correct annotation and full-length protein domain were selected for subsequent study (Table 3). The specific amplification and expression data were obtained through real-time PCR; histone H3 (CL15850.Contig2_F0463-PN) was defined as the most suitable internal control gene of black pepper (Fig 4). In addition, large amounts of SSR markers and primers were identified from the transcriptome data (S3 Table). The polymorphisms of 24 pairs of primers were randomly determined by denaturing polyacrylamide gel. Approximately half of the polymorphisms could be used in related studies. To our knowledge, the polymorphic SSR markers are important molecular tools, which have been extensively utilized for genetic diversity, gene mapping, molecular breeding, and gene-based association studies [38]. Our study on internal control gene and polymorphic SSR marker identification not only illustrates the usefulness of our transcriptome data but also provides available reference data for further research.

Black pepper is well known for its specific alkaloids in fruits. The most important functional substance among piperidine alkaloids was piperine, which is accounted for the pungent taste and the medicinal effect of black pepper [25,39]. We described the transcriptome of black pepper fruit to investigate piperine biosynthesis. As a lysine-derived alkaloid, piperine undergoes several steps, including lysine decarboxylation as the first step in alkaloid biosynthesis in which cadaverine is produced [40]. Cadaverine is then used as a functional substance to synthesize piperidine through a series of reactions, including oxidation, dehydration, and cyclization [41–43]. A previous study showed that piperidine is the direct precursor of piperine synthesis [12]. Although we determined the main line of the piperine synthetic pathway from different research results, a systemic study on piperine synthesis and key gene identification is limited. In KEGG enrichment, potential genes involved in lysine metabolism were initially selected. Based on the overall consideration of annotation and sequence information, the unigenes showed consistent functional description. The full-length protein domain is shown in Table 2. The genes presented in this study would provide putative targets for further research on piperine synthesis.

## Conclusion

This study presents the first transcriptome sequencing analysis of black pepper fruit using Illumina RNA-Seq technology. A total of 44,061 unigenes with an average length of 1,345 nt were generated and 40,537 were annotated. Based on these annotated unigenes, the characteristic of the transcriptome was illustrated comprehensively by bioinformatics analysis. The subsequent work of lysine metabolism related gene, internal control gene and polymorphic SSR identification suggest the availability of transcriptome data. Our study highlight the potential of RNA-seq for functional genomics researches on different species which genomic sequence data are not available. All of these data provide fundamental reference for further functional genomics studies on black pepper.

## Supporting Information

S1 Table Whole annotation information of the transcriptome data. (XLSX)

S2 Table Detailed subcategories of KEGG annotation. (XLS)

S3 Table Detailed results of SSR detection. (XLSX)

S4 Table Results of SSR primer polymorphism analysis. (DOC)
