# Correction: *De Novo* Assembly and Characterization of Fruit Transcriptome in Black Pepper (*Piper nigrum*)

**DOI:** 10.1371/journal.pone.0136028

**Published:** 2015-08-14

**Authors:** Lisong Hu, Chaoyun Hao, Rui Fan, Baoduo Wu, Lehe Tan, Huasong Wu

There are errors in the Funding section. The correct funding information is as follows: This project was funded by the Ministry of Agriculture of the People’s Republic of China (2015NWB046) and the Program for the Top Young Talents in Chinese Academy of Tropical Agricultural Sciences (1630052015049). The funders had no role in study design, data collection and analysis, decision to publish, or preparation of the manuscript.
