# Supplementary material for: De Novo Assembly and Characterization of Fruit Transcriptome in Black Pepper (Piper nigrum)
Source: PLoS One. 2015 Jun 29;10(6):e0129822. doi: 10.1371/journal.pone.0129822 (PMC4488137; doi:10.1371/journal.pone.0129822)
Supplement: S4 Table — (DOC) [file pone.0129822.s004.doc]

24 SSR primers in polymorphism analysis:

| Gene ID (1-24) | Forward primer (5`-3`) | Reverse primer (5`-3`) |
| --- | --- | --- |
| CL10038.Contig1_F0463-PN_1956_5 | CCGACGCGAACTTCTTCAT | CATAAAATCGAGGAAAAGCGAG |
| CL1003.Contig2_F0463-PN_1617_1 | GTACCACGACTTGCGAAGG | TCAAATCTTTACCATTTCCCTCC |
| CL10084.Contig2_F0463-PN_1961_1 | AAAACGCAATATACCCAAAAGGT | GTCGGAACCCCGAAGCAG |
| CL10087.Contig2_F0463-PN_1288_1 | GTAGTCGACGGAAAAGGGGT | CCTTCTTCCTTTCACTTCCTTGT |
| CL10172.Contig2_F0463-PN_1280_1 | GTACGCTTCACAGTACCATGTTG | AGTCTCTGGCAATCACAACAACT |
| CL10176.Contig2_F0463-PN_1156_1 | TGAAGAATGGATAAGGATCTGGA | ATGATTTACTTGTTCAGGTGGGA |
| CL10451.Contig2_F0463-PN_739_1 | GCAAGAAAAATCAAGAAACATGG | GGATGAATGAGCTTGAGGATAAA |
| CL1046.Contig1_F0463-PN_1908_1 | AAGAAAGGAGACCAAATCTCCAC | GCTCGTTTACATTAAAGCTCGAC |
| CL10518.Contig1_F0463-PN_1590_1 | GACTTCAACACGATCAGACCC | AAGTTGGGATCTTTGCGACTAAT |
| CL10564.Contig3_F0463-PN_125_1 | TGGTTTAATGGCTAAGAAAACGA | CATACTACGGGGTAAAACACCAA |
| CL10615.Contig2_F0463-PN_326_2 | CTGTGCTTGTTGCTGTTGTATCT | TTACTGCATGGTCTCATCAGTTG |
| CL10717.Contig2_F0463-PN_780_1 | GATGAGGATGATTGGGAGGAT | CAATCTTGTACAGCCCATAAAGC |
| CL10739.Contig1_F0463-PN_2020_1 | GTTCTTCAGTCCAGACTCAGCAT | AGGAAGAAAACTTTGCGAGTCAA |
| CL10785.Contig1_F0463-PN_1435_1 | CATAAACGAAACCCAAAAGAACA | CAGTTCGAGATCTCCTACGAGTG |
| CL10878.Contig2_F0463-PN_821_1 | TCATTACCATGAGCACTCTTTCT | CCCCCTTTCTGTAAGACAAAACT |
| CL10947.Contig1_F0463-PN_779_1 | TCTCTCTTCCAAACTGCAAAAAG | TGCAGACTCATTCATGTGATTTC |
| CL10969.Contig2_F0463-PN_1770_1 | GAGTACGAGGAGTACGACGAGGT | ATCTCCTTGAGCCTCTTCTTCAT |
| CL10974.Contig1_F0463-PN_1450_1 | ACTACGACTTTCACCTCCACTCC | TCTTGAAGGAGCAGGTCAGATAG |
| CL11035.Contig2_F0463-PN_1355_1 | CTCTTCAAGCCCCTCTCCAC | GGGTTCCACTAGTTCTGGGG |
| CL11143.Contig2_F0463-PN_1165_1 | AGTAACATTAAGCTCCTGCAACG | ATCTTCTCCTTCAACTCCAAAGG |
| CL1117.Contig1_F0463-PN_1302_1 | GCTTTCAAAGGACTTACTGGGTT | ACATCTGATGAAGGGTGATCCTC |
| CL11225.Contig1_F0463-PN_1005_1 | ATGGAGTGCAATTCTAGCTCAAC | GACGGTCCACTTTAGTCAATCTG |
| CL11232.Contig2_F0463-PN_375_1 | TAGCCATTCTAGGCAATAAGCTG | CGCCTTATTAAAGACCAAACACA |
| CL11272.Contig1_F0463-PN_242_1 | TACACGTTATTGCTGTCTTCCCT | ACCTGGCGGATAAGAGGGT |


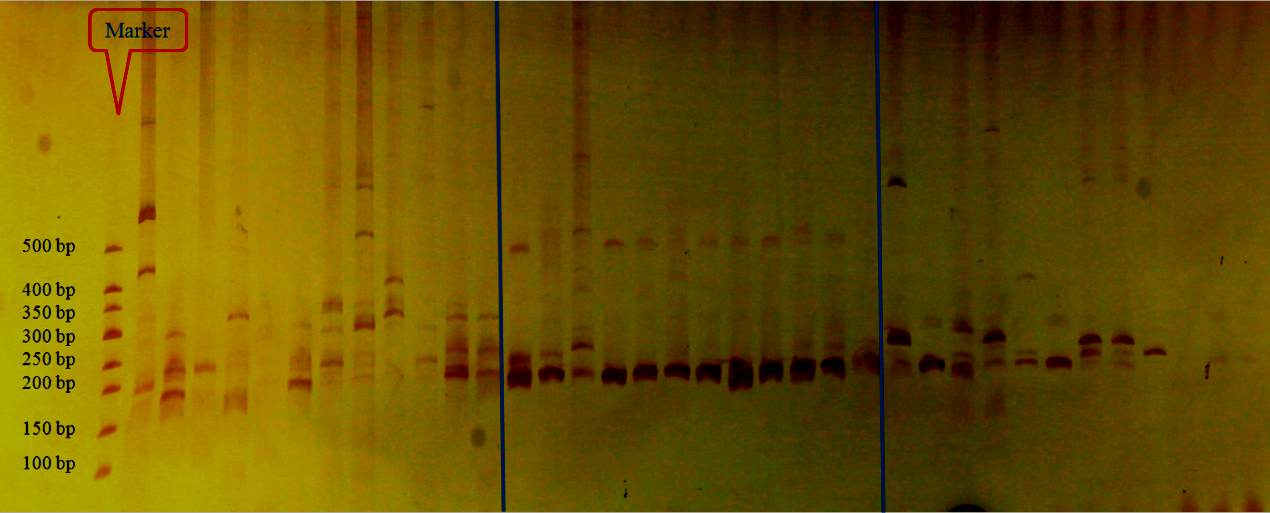


The typical result in polymorphism analysis:

Each 12 lane mean one pair primer, which separated with the blue line. The order of 12 Piperaceae species was followed: 1.*Piper nigrum* cv. PC036, 2.*Piper nigrum* cv. PC003, 3.*Piper longum*, 4.*Piper laetispicum*,5.*Piper hancei* -1, 6.*Piper hancei* -2, 7.*Piper wallichii*, 8.*Piper umbellatum*, 9.*Piper bavinum*,10.*Piper callosum*, 11.*Piper nigrum* cv. Aman semmeggoh, 12.*Piper thomsonii*
